# Growth Hormone Receptor Gene Expression Increase Reflects Nutritional Status Improvement in Patients Affected by Crohn's Disease

**DOI:** 10.3389/fped.2018.00338

**Published:** 2018-11-12

**Authors:** Sara Pagani, Elena Bozzola, Caterina Strisciuglio, Cristina Meazza, Erasmo Miele, M. Malamisura, Paola De Angelis, Mauro Bozzola

**Affiliations:** ^1^Unit of Pediatrics and Adolescentology, Department of Internal Medicine and Therapeutics, University of Pavia, Pavia, Italy; ^2^Pediatrics Department Bambino Gesù Children's Hospital, Rome, Italy; ^3^Department of Women, Children and General and Specialized Surgery, Second University of Naples, Naples, Italy; ^4^Pediatric Gastroenterology Division, University of Naples Federico II, Naples, Italy; ^5^Digestive Surgery and Endoscopy Unit,Bambino Gesù Children's Hospital, Rome, Italy

**Keywords:** IGF-I, short stature, inflammation, GHBP, growth hormone receptor, Crohn's disease

## Abstract

**Background:** We proposed to verify the role of growth hormone receptor gene expression in growth failure of children with Crohn's disease (CD).

**Methods:** We measured serum levels of growth hormone binding protein (GHBP) and insulin-like growth factor-I (IGF-I), and growth hormone receptor (GHR) gene expression in peripheral blood mononuclear cells of 21 patients with CD (before and after therapy) and in 27 age-sex-matched controls.

**Results:** At diagnosis, significantly lower insulin-like growth factor-I and growth hormone binding protein levels were found in the CD group compared to the controls. Growth hormone receptor mRNA expression was lower in patients at diagnosis compared to the controls, even though the difference did not reach statistical significance, and significantly increased in patients in the following year. Insulin-like growth factor-I levels showed significant improvements 1 year after diagnosis compared to basal levels. On the contrary, growth hormone binding protein values had not significantly changed after 1 year of therapy.

**Conclusion:** Our study raises the hypothesis of another mechanism through which cytokines interact with the growth hormone/insulin-like growth factor-I (GH/IGF-I) axis.

## Introduction

Crohn's disease (CD) is a chronic inflammatory bowel disease (IBD) that can affect the entire gastrointestinal tract (1). Although CD can develop at any age, many patients are diagnosed in childhood; specifically, ~20% of patients with CD develop IBD before the age of 20 (2).

Malnutrition and impaired growth are the major complications of pediatric CD (1) and approximately one-third of CD children experience linear growth retardation (3). Abnormalities of linear growth during childhood probably lead to reduced adult stature in CD patients Indeed, up to 25% of CD patients do not reach their final target height (4). These complications, which may precede clinical evidence of CD by a few years, are more frequent in CD children compared to those with Ulcerative Colitis (UC), both at diagnosis and during follow-up (5, 6). Therefore, progressive growth failure is an important feature of disease severity and can be used to monitor the success of medical treatment and nutritional or surgical intervention (5). The causes of growth failure in these patients are complex and include the inflammatory process itself, malnutrition, malabsorption, and treatment with glucocorticoids. These factors act systemically as well as at growth plate level to inhibit bone growth.

The GH-IGF I axis is the major regulator of longitudinal bone growth: the systemic actions of the growth hormone (GH) are mediated by IGF-I, which is produced by the liver. Furthermore, GH has been demonstrated to act, at the epiphyseal growth plate, either directly by stimulating resting/stem-like chondrocytes proliferation, or indirectly, through IGF-I, to promote chondrocyte hypertrophy (7). Levels of circulating IGF-I and its binding protein (IGFBP3) are good indicators of peripheral GH action, while decreased serum IGF-I and IGFBP3 levels are found in active CD (8).

Since the process of growth is supported by an adequate supply of nutrients, chronic malnutrition has long been implicated as a cause of poor growth in CD patients (9). Optimizing nutrition is thus an important step in managing growth failure, and the degree of intervention can be adjusted according to the severity of the condition. However, growth retardation can also occur under adequate nutritional conditions or even in absence of weight loss, indicating that other factors may be present (10). For example, an increase in cytokine production, induced by chronic inflammation, may affect growth, such as in infection and active systemic juvenile chronic arthritis (11–13). These inflammatory factors induce a state of GH resistance through different mechanisms: down-regulation of the growth hormone receptor (GHR), up-regulation of post receptor inhibitory proteins, reduced protein synthesis and/or increased protein degradation. Inflammatory cytokines have not only systemic actions but also direct local effects at the growth plate level; for example TNF-α and Interleukin-1β (IL-1β) have been found to increase chondrocyte death (7). These mechanisms decrease circulating concentrations of IGF-I in CD patients despite normal GH secretion, resulting in linear growth failure (14). Finally, in CD patients, therapy with glucocorticoids (GCs) suppress the GH/IGF-I axis by inhibiting GH secretion (15) and downregulating the GH receptor in the liver, thereby inhibiting IGF-I secretion and activity (16). Furthermore, GCs inhibit chondrocyte proliferation, as well as increase their apoptosis (17). Thus, GCs may have positive effects in patients with chronic inflammation through their suppression of cytokine levels (18), but long-term GC therapy may lead to severe negative side effects such as osteoporosis and growth impairment.

To verify whether growth hormone receptor (GHR) gene expression plays a role in the growth failure of children with CD, as a result of the chronic inflammatory condition and malnutrition, we measured GH binding protein serum levels and GHR gene expression in lymphocytes from peripheral blood of patients with CD and from age-sex-matched controls. In CD patients, we analyzed the same parameters 1 year after onset of the disease and correlated them with anthropometric data and clinical activity, in order to discover whether these parameters may be influenced by therapy.

## Patients

In this multicenter study, 21 patients (11 males and 10 females) with a mean age of 12.1 ± 0.9 years were enrolled at the Bambino Gesù Children's Hospital in Rome and at the Pediatric Gastroenterology Division Unit of the University of Naples hospital. Subjects were consecutively recruited over 12 months and were evaluated prospectively for 1 year. Diagnosis of CD was based on clinical history, physical examination, radiologic studies, endoscopic appearance, and histological findings, according to the revised Porto criteria (19). Patients were classified, according to the Paris classification (20), as: 4 (A1,L2,B1); 1 (A1L1/L3B1); 5 (A1,L1,B1); 2 (A1L3/L4B1); 1 (A1b, L4a,B1); 2 (A1,L3B2); 4 (A1,L3,B1); 1 (A1, L2/L4, B1); 1 (A2,L1,B2).

Anthropometric measurements were taken by a professional nurse and recorded in the clinical diary, in accordance with World Health Organization guidelines. Stature was measured with a stadiometer in patients standing barefoot. Height was expressed according to chronological age, as a standard deviation score.

Patients had both elevated levels of both C reactive protein (CRP average: 31.69 mg/dl, normal value: < 0.05 mg/dl) and of erythrocyte sedimentation rate (ESR average: 35.33 mm/h, normal value < 20 mm/h) at disease onset. Range of CRP and ESR variations are showed in Table 1.

**Table 1:** Inflammation and PCDAI values in CD patients at the time of diagnosis (t0) and at 1-year follow-up.

	**CD T0 (min-max)**	**CD T1 (min-max)**	**Normal values**
CRP (mg\dl)	0.28–165.05	< 0.33–11.3	< 0.05
ESR (mm\h)	4–77	1–40	< 20
PCDAI	12.5–60	0–22.5	< 10

The common presenting symptoms were abdominal pain (6/21), diarrhea (11/21), weight loss (9/21), malnutrition (5/21), rectorrhagia (5/21), and extraintestinal manifestations (10/21, including anemia 4/21, oral ulcers 4/21, and erythema nodosum 2/21). Perianal lesions were present in 4/21 patients (including anal skin tags 2/21, perianal fissures 1/21, and perianal fistulas 1/21).

Pediatric Crohn's Disease Activity Index (PCDAI) was determined according to Valletta et al. (21), with clinical remission defined as a PCDAI score of <10 and moderate/severe CD with a PCDAI of >30. At the time of diagnosis, 10 patients had a PCDAI ≥30, revealing moderate/severe activity. Range of variation of PCDAI values is showed in Table 1.

Blood was collected on the same day as the baseline clinical evaluation and after 1 year of therapy to measure IGF-I, GHBP and GH-R gene expression. Regarding therapy, at diagnosis patients were prescribed: mesalazine (Asalex, Chiesi Pharmaceuticals or Pentasa, Ferring; 50%), azathioprine (Azatiioprina, Aspen Pharma or Azatioprina, Sandoz; 14%), enteral nutrition (Modulen IBD, Nestlè; 50%), oral steroids (Deltacortene, Bruno Pharmaceuticals; 50%), nutritional treatment (Modulen IBD, Nestlè; 38%), nine-month infliximab therapy (Emicade, MSD Italia; 1 patient) and 6-month adalimumab therapy (Humira, Abbvie srl; 1 patient).

At follow up, a decrease was found in both CRP and ESR (2.4 mg/dl and 9.5 mm/h, respectively). At this time, the average PCDAI score was 6.5.

Twenty-seven healthy children, sex- and age-matched (mean age: 9.4 ± 0.27 years), were recruited as a control group at the Pediatric Auxology Unit of the Fondazione IRCCS Policlinico San Matteo Hospital. Their mean height was −0.66 ± 0.03 SDS and their mean Body Mass Index (BMI) was −0.8 ± 0.19 SDS (World Health Organization standards, 2006).

Written informed consent was obtained from parents or legal guardians of all children, and patients older than 13 signed a statement of assent. The study was approved by the Ethics Committee of the centers involved in the study.

## GHBP evaluation

Serum levels of GHBP were measured by a commercially available ELISA (DSL-10-48100 ACTIVE hGHBP Elisa-Webster, Texas, USA). The minimum detectable concentration was 1.69 pmol/l. The intra- and inter-assay coefficients of variation were 5.59–4.78% and 8.36–5.11%, with a quality control range of 20.25–198.24 pmol/l and 19.99–195.78 pmol/l, respectively.

## IGF-I determination

The serum IGF-I concentration was measured by an automatic assay that utilizes a solid-phase, enzyme-labeled chemiluminescent immunometric assay (Immulite 2 000 IGF-I-DPC, Los Angeles, CA and Immulite Analyzer). The intra-assay coefficients of variation were 3.9–2.4%, with a quality control range of 77–1,358 ng/ml. IGF-I values are expressed as mean a standard deviation score (SDS) according to Elmlinger et al. (22).

## GHR gene expression

Peripheral blood mononuclear cells (PBMC) of patients and age-matched controls were separated by Ficoll density gradient centrifugation using a standard procedure (centrifugation at 1,800 rpm for 30 min at room temperature, followed by the recovery of the PBMC ring at the interface).

For Real-Time GHR gene expression analysis, total RNA was isolated from PBMC using RNAeasy mini-columns (Qiagen, Hilden, Germany). RT-PCR was carried out with the SuperScript First-Strand Synthesis System. An RNA/primer mixture was prepared containing total RNA, oligo dT (50 ng/μl), 10mM dNTP mix and DEPC water. The samples were incubated at for 5 mins and then on ice for at least 1 min. A master reaction mixture, containing 10X RT buffer, 25 mM MgCl_2_, 0.1 M DTT, and RNAase OUT, was prepared for each sample. The reaction mixture was then added to the RNA/primer mixture, samples were mixed briefly and kept at room temperature for 2 min. Fifty units of SuperScript II RT was added to each tube, the samples were mixed and incubated at for 10 mins, and the tubes were then incubated at for 50 mins, heat inactivated at for 15 mins, and chilled on ice. First strand cDNA was stored at −20°C until use for real-time PCR. Quantitation of GHR mRNA expression was determined by quantitative real-time RT-PCR (Real-Time PCR 3500-Applied Biosystems) and assays on demand were used (Hs00174872_m1 Applied Biosystems). Normalization and validation of the data were carried out using glyceraldehyde-3-phosphate dehydrogenase (GADPH) as a housekeeping control gene. Each GHR or GAPDH probe was labeled with a fluorescent reporter (FAM. Specifically, a 25 microliter volume reaction mixture containing 1.25 μl Assay, 12.5 μl Master Mix, 10.25 μl H2O, and 1 μl cDNA was treated under the following conditions: for 10 mins, for 15 s, for 1 min, for 40 cycles.

Quantitative Real-time PCR data were calculated by a standard curve and expressed as attograms GHR/(5 × 10^5^) attograms GAPDH. We chose the GAPDH gene because its expression shows reproducible and stable levels, moreover GAPDH CT are in the correct range for comparisons with the GHR gene. Furthermore, we evaluated possible variations in GAPDH expression values before considering them suitable for GHR gene expression data normalization and if necessary, disregarded the results.

The amplification efficiency of GHR with regard to GAPDH mRNA expression was evaluated by analyzing the ΔCt variation with template dilutions in the 1,000-fold range.

## Statistical analysis

Data are expressed as the mean ± SEM. Statistical differences between patients before therapy and controls were determined using the Mann-Whitney *U*-test (Medcalc Software); whereas the non-parametric Wilcoxon test for paired samples was used to compare values in patients before and after 1 year of treatment. Correlations were analyzed using the Spearman's rank correlation test.

A value of *p* < 0.05 was considered statistically significant.

## Results

**Figure 1:**
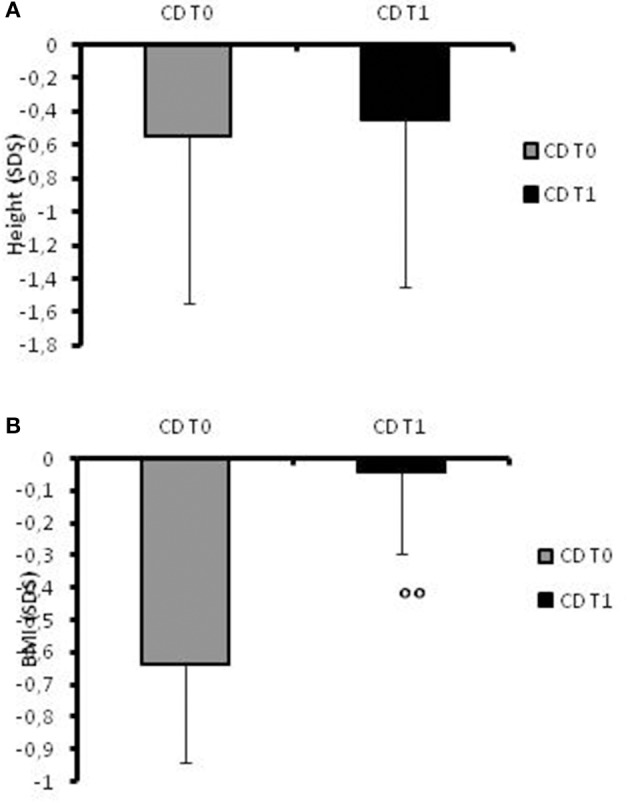
**(A)**Height SDS values in CD patients at the time of diagnosis (t0) and at 1-year follow-up. **(B)** BMI SDS values in CD patients at the time of diagnosis (t0) and at 1-year follow-up. °°**:** *p* = 0.03 CD children at t0 vs. CD children at t1.

At recruitment, CD children presented a mean height of −0.55 ± 0.3 SDS (Figure 1A) and in two cases, the stature was below −2SD. Their BMI mean value was −0.64 ± 0.3 SDS (Figure 1B).

**Figure 2:**
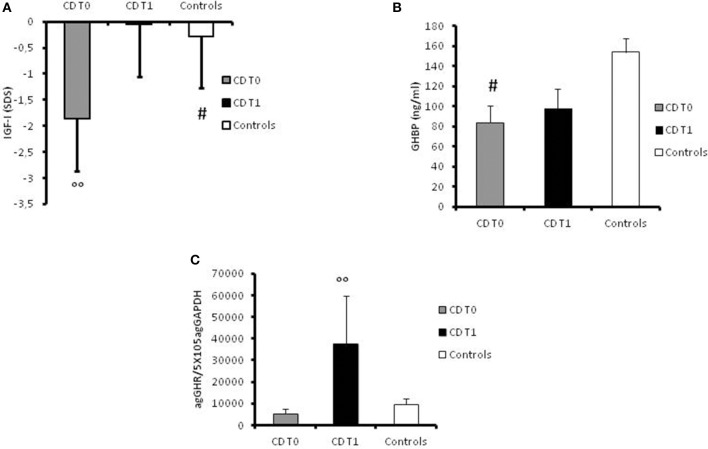
**(A)** Serum values of IGF-I in controls, in CD patients at the time of diagnosis (t0) and at 1-year follow-up, expressed as ng/ml. **#:** *p* = 0.007 CD children vs. controls. °°**:** *p* < 0.039 CD children at t0 vs. CD children at t1. **(B)** Serum value of GHBP in controls, in CD patients at the time of diagnosis (t0) and at 1-year follow-up, expressed as ng/ml. **#:** *p* < 0.05 CD children vs. controls. **(C)** GHR gene expression values in controls, in CD patients at the time of diagnosis (t0) and at 1-year follow-up, expressed as agGHR/5 × 10^5^agGAPDH. °°**:** *p* = 0.033 CD children at t0 vs. CD children at t1.

At disease onset, significantly (*p* = 0.007) lower levels of IGF-I were found in the CD group (mean value −1.87 ± 0.91 SDS) compared to the control group (mean level −0.28 ± 0.14 SDS; Figure 2A). GHBP was also significantly (*p* < 0.05) lower in patients compared to controls (83.75 ± 17.28 ng/ml and 153.95 ± 13.61 ng/ml, respectively; Figure 2B).

Furthermore, GH-R mRNA expression was lower in patients at diagnosis (5201.59 ± 2393.74 agGHR/5 × 10^5^agGAPDH) compared to controls (9515.06 ± 2605.68 agGHR/5 × 10^5^agGAPDH) albeit not statistically significant (Figure 2C).

At follow-up, the mean height increased to −0.45 SDS (range −1.76 to +1.24; Figure 1A) and the patients' BMI SDS significantly (*p* = 0.03) increased after 12 months of therapy (−0.045 ± 0.25) compared to the onset of disease (Figure 1B). None of the patients had a stature lower than −2 SD at the 1-year follow-up.

However, the most important result was the significant (*p* = 0.033) increase in GHR mRNA expression in all patients at 1-year follow-up (37572.17 ± 22135.23 agGHR/5 × 10^5^agGAPDH**)** in comparison with basal values (Figure 2C). Moreover, IGF-I levels had significantly (*p* = 0.039) improved in the year following diagnosis (−0.051 ± 0.2 SDS) compared to levels at disease onset (Figure 2A). On the contrary, GHBP values did not significantly change after 1 year of therapy (98.18 ± 19.62 ng/ml) compared to basal levels (Figure 2B).

## Discussion

The inflammatory process in CD patients has been associated with the pathogenesis of growth delay through both IGF-I and GH-dependent mechanisms mediated by cytokines, although other factors such as innate genetic variations and inadequate caloric intake are implicated in GH resistance with consequent negative impact on linear growth (6). In CD patients, nutritional and pharmacological support, especially after the introduction of biological agents, decreases mucosal inflammation and alleviates symptoms, ameliorating linear growth and pubertal development. Indeed, the presence of lower levels of inflammatory cytokines in CD has been correlated with an increase in height growth velocity (7).

GH acts through the GHR, which is most commonly expressed in the liver, but also in the growth plate of long bones, in the intestinal epithelium and in all the layers of the gastrointestinal wall (23).

CD children show normal GH secretion in contrast with low circulating IGF-I levels, suggesting a significant degree of resistance to the effects of GH with consequent impaired linear growth. Hepatic GH resistance is induced by cytokines through two main mechanisms: down-regulation of GHR and up-regulation of the members of the SOCS (suppressor of cytokine signaling) family. In particular, cytokines such as Interleukine-6 (IL-6) increased SOCS3 expression, reducing STAT5b activity (24).

We wanted to verify whether GHR gene expression plays a role in the growth failure of children with CD, as a result of the chronic inflammatory condition and malnutrition.

In our study, similar to previous reports, the IGF-I level was significantly lower in CD patients compared to controls, consistent with GH resistance (13). At 1-year follow up, the patients' IGF-I value had significantly increased, reaching higher levels compared to control values. Specifically, the IGF-I increase was significant and was concomitant with an overall improvement as assessed by physical examination of the patients. This probably reflects the reduced inflammation and the improvement of nutritional status during disease control. The BMI of our patients had also significantly increased after 1 year of therapy.

Cell surface amounts of GHR are the most important determinant of GH responsiveness (25). In our patients, GHR mRNA levels were lower at diagnosis consistent with an inflammatory state, although this result was not statistically significant. One year later, when the disease was under control, the GHR values were found to have significantly increased, together with the IGF-I levels. On the contrary, basal values of GHBP in CD patients were significantly lower compared to controls but they did not increase after 1 year of therapy. Therefore, our data show that GHBP level and GHR function are not closely correlated, as demonstrated also in other physiological and pathological conditions (26). GHR regulation and its cleavage to GHBP are tissue-specific. Therefore, it is possible that, in our patients, these mechanisms lead (27) to an increase in GHR availability on the cell's surface, thus improving GH action.

To the best of our knowledge, there are no previous studies reporting GHR gene expression values in CD children at onset of the disease and during follow-up. Our study raises the hypothesis of another cytokine interaction with the GH/IGF-I axis. This mechanism involves the GHR before the start of signal transduction. Since GHR is present at growth plate level, it is possible of course that there is not only systemic but also local GH/IGF-I resistance.

However, in our study only two patients showed severe growth retardation at diagnosis with a height under −2 SDS (9.5%) and this value is lower than the rate that has been previously reported (28). This is possibly due to the shorter interval between the onset of symptoms and diagnosis, due to improvements in diagnosis as shown also in the study of Song et al. (1). In our study, the median interval from onset of symptoms to diagnosis was 5.5 months, whereas the median diagnostic delay was 11.7 months in the previous study (29). Indeed, it has been shown that (30) the duration of developmental delays and height in CD patients are significantly correlated, suggesting that earlier diagnosis might minimize and prevent growth retardation by reducing the duration of symptoms before treatment, and so rectifying the growth inhibiting effects of malnutrition and inflammatory cytokines.

Our study has some limitations. Firstly, we acknowledge that the sample size was small, secondly the heterogeneity in drug treatments. Further studies with a larger sample size may provide more precise results.

Serum IGF and GHR gene expression levels change during the course of therapy, according to the clinical features, suggesting that disease activity modification is accompanied by variations in hypothalamic/pituitary-related factors. During disease remission, IGF-I and GHR mRNA expression increase, supporting the possibility of a strong correlation between growth factors, inflammatory process and nutritional status. These findings underline the importance of prompt control of inflammatory activity in order to prevent stunted growth and to promote good overall outcomes in mild to moderate CD. Furthermore, monitoring CD patients' growth is indispensable in order to assess their response to therapy over time (31).

## Data availability

The raw data supporting the conclusions of this manuscript will be made available by the authors, without undue reservation, on request of any qualified researcher.

## Ethics statement

Written informed consent was obtained from parents or legal guardians of all children, in accordance with the Declaration of Helsinki; patients older than 13 years signed a statement of assent. The study was approved by the Ethics Committee at both the Bambino Gesù Hospital and San Matteo Foundation Hospital.

## Author contributions

MB and PD designed the study, revised the manuscript, and approved its final version. SP and CM performed experiments, collected, and analyzed data. SP and EB wrote the manuscript. CS, EM, and MM gave technical support and conceptual advice. All authors read and approved the final manuscript.

### Conflict of interest statement

The authors declare that the research was conducted in the absence of any commercial or financial relationships that could be construed as a potential conflict of interest.

## Abbreviations

CD: Crohn's disease
GHBP: growth hormone binding protein
IGF-I: insulin-like growth factor-I
IBD: inflammatory bowel disease
UC: ulcerative colitis
GH: growth hormone
IGFBP3: IGF binding protein
TNF-α: tumor necrosis factor α
GHR: growth hormone receptor
IL-1β: Interleukine-1β
GCs: glucocorticoids
PCDAI: Pediatric Crohn's Disease Activity Index
SOCS: suppressors of cytokine signaling
IL-6: Interleukine-6.
